# Multigene Knockout Utilizing Off-Target Mutations of the CRISPR/Cas9 System in Rice

**DOI:** 10.1093/pcp/pcu154

**Published:** 2014-11-11

**Authors:** Masaki Endo, Masafumi Mikami, Seiichi Toki

**Affiliations:** ^1^Plant Genome Engineering Research Unit, Agrogenomics Research Center, National Institute of Agrobiological Sciences, 2-1-2 Kannondai, Tsukuba, Ibaraki, 305-8602 Japan; ^2^Graduate School of Nanobioscience, Yokohama City University, 22-2, Seto, Kanazawa-ku, Yokohama, Kanagawa, 236-0027 Japan

**Keywords:** CDK, CRISPR/Cas9, Off-target mutation, Rice

## Abstract

The clustered regularly interspaced short palindromic repeat (CRISPR)-associated endonuclease 9 (CRISPR/Cas9) system has been demonstrated to be a robust genome engineering tool in a variety of organisms including plants. However, it has been shown that the CRISPR/Cas9 system cleaves genomic DNA sequences containing mismatches to the guide RNA strand. We expected that this low specificity could be exploited to induce multihomeologous and multiparalogous gene knockouts. In the case of polyploid plants, simultaneous modification of multiple homeologous genes, i.e. genes with similar but not identical DNA sequences, is often needed to obtain a desired phenotype. Even in diploid plants, disruption of multiparalogous genes, which have functional redundancy, is often needed. To validate the applicability of the CRISPR/Cas9 system to target mutagenesis of paralogous genes in rice, we designed a single-guide RNA (sgRNA) that recognized 20 bp sequences of cyclin-dependent kinase B2 (*CDKB2*) as an on-target locus. These 20 bp possess similarity to other rice CDK genes (*CDKA1*, *CDKA2* and *CDKB1*) with different numbers of mismatches. We analyzed mutations in these four CDK genes in plants regenerated from Cas9/sgRNA-transformed calli and revealed that single, double and triple mutants of *CDKA2*, *CDKB1* and *CDKB2* can be created by a single sgRNA.

## Introduction

The type II clustered regularly interspaced short palindromic repeat (CRISPR)-associated endonuclease 9 (CRISPR/Cas9) system (Sorek et al. 2013) is a novel molecular tool for site-specific genome modification (Jinek et al. 2012, Cong et al. 2013, Cho et al. 2013, Hwang et al. 2013, Jiang et al. 2013, Jinek et al. 2013, Mali et al. 2013). In plants, three short reports in 2013 described the first applications of the CRISPR/Cas9 system to plant genome engineering (Li et al. 2013, Nekrasov et al. 2013, Shan et al. 2013). Since then, many successful reports have followed (for a review see Belhaj et al. 2013). The specificity of CRISPR/Cas9-mediated DNA cleavage is determined by the 20 bp sequence in the single-guide RNA (sgRNA) and the NGG trinucleotide known as the protospacer-adjacent motif (PAM) that is recognized by Cas9 (Jinek et al. 2012). The ‘seed region’ of approximately 12 bases proximal to the PAM motif is the most crucial for pairing and DNA cleavage, while mispairing in the distal bases can sometimes be tolerated (Fu et al. 2013). Furthermore, three research groups showed independently that CRISPR/Cas9 indeed induces off-target mutations, even at sites that differ by five nucleotides from the on-target site in human cells (Fu et al. 2013, Hsu et al. 2013, Pattanayak et al. 2013). Off-target mutation is a critical issue in the field of gene therapy because such mutations run the risk of leading to alternative disease states. On the other hand, in the case of seed-propagated plants, off-target mutations can be removed by backcrossing. Furthermore, such off-target mutations may be a potentially effective tool for multigene knockout of homeologous and paralogous genes. Because many plant species are polyploid, disruption of multihomeologous gene arrays is often needed to obtain a desired phenotype. Autopolyploids have several copies of the same or near identical genomes. On the other hand, allopolyploids typically have two or more distinct subgenomes. Furthermore, polymorphisms exist between cultivars; thus, obtaining complete genome information and designing perfectly matched sgRNAs to each target sequence is difficult in many situations in plant breeding. Even in diploid plants, genomes of allogamous plants are heterogenic and extensive polymorphisms exist. To confirm the possibility of achieving multiparalogous gene knockout by utilizing off-target mutations via the CRISPR/Cas9 system in rice, we selected 20 bp in the cyclin-dependent kinase B2 (*CDKB2*) gene as a target sequence for the sgRNA. Like animals, plants have multiple CDK-related protein kinases, which are classified into six types: CDKA–CDKF (Joubès et al. 2000). A-type CDK (CDKA) is closely related to yeast Cdc2 (*Saccharomyces cerevisiae*)/Cdc28 (*Schizosaccharomyces pombe*), and is expressed throughout the cell cycle (Colasanti et al. 1991, Ferreira et al. 1991, Hirt et al. 1991, Hirt et al. 1993, Fobert et al. 1996). Rice CDKA is classified into two subtypes: CDKA1 (Os03g0118400) and CDKA2 (Os02g0123100). The other major CDK in plants is B-type CDKB. CDKB is plant specific and further classified into two subtypes: CDKB1 (Os01g0897000) and CDKB2 (Os08g0512600). Expression of CDKB is under strict cell cycle control; CDKB1 is expressed from late S to M phase, while CDKB2 is expressed from G_2_ to M phase (alfalfa, Magyar et al. 1997; rice, Umeda et al. 1999; tobacco, Porceddu et al. 2001; Arabidopsis, Menges et al. 2005). The expression pattern during the cell cycle differs for CDKAs and CDKBs, but they share homology in amino acid sequences and in the corresponding coding DNA sequences. Thus, in the present study, we exploited these latter features to examine the possibility of multi-CDKA and CDKB gene knockout by taking advantage of off-target mutations mediated by CRISPR/Cas9.

## Results

### Selection of target sequence

**Fig. 1:**
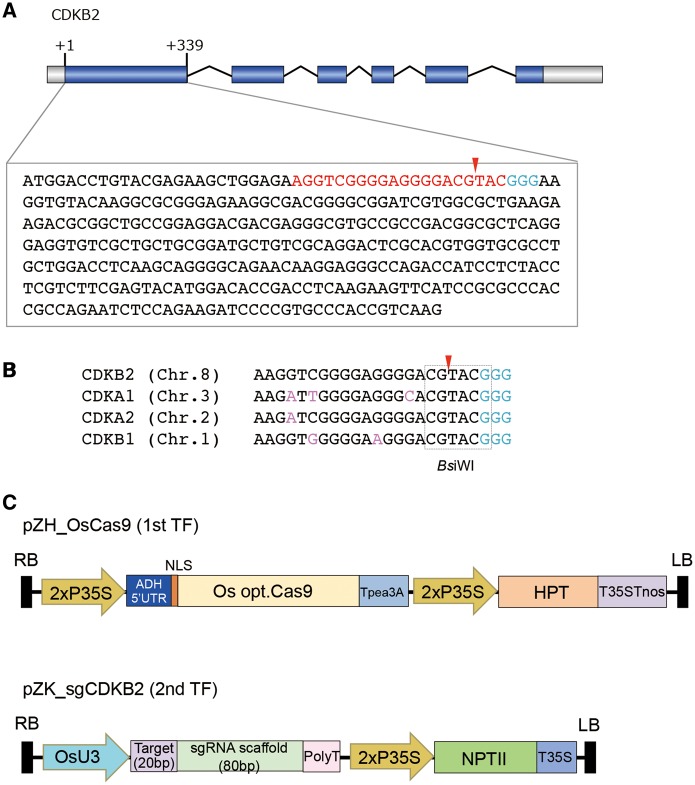
Schematic representation of CRISPR/Cas9-mediated target mutagenesis in this study. (A) Target site of CRISPR/Cas9-mediated target mutagenesis in the rice *CDKB2* gene. The PAM sequence (NGG) is shown in blue and the 20 bp target sequence is shown in red. The red arrowhead indicates the expected cleavage site. (B) Homology of target sequences in *CDKA* and *CDKB* genes. Mismatches to the target sequence on *CDKB2* are shown in pink. The dotted box indicates the *Bsi*WI recognition sequence (CGTACG). (C) Vector constructs used in this study. pZH_OsCas9 was used for the first transformation and pZK_sgCDKB2 was used for the second transformation.

Rice CDKA and CDKB2 sequences share homology in amino acid sequence and the corresponding coding DNA sequences (Supplementary Fig. S1). We chose 20 bp in the first exon of the *OsCDKB2* gene as a target sequence for CRISPR/Cas9 (Fig. 1A). A 20 nucleotide region at the 5′ end of the PAM of *CDKB2* has homology to the corresponding 20 nucleotides in *CDKA1*, *CDKA2* and *CDKB1* with different numbers of mismatched bases (Fig. 1B; Supplementary Fig. S2).

### Vector construction for the CRISPR/Cas9 system

To apply the CRISPR/Cas9 expression system to *Agrobacterium*-mediated transformation in rice, we synthesized a *Streptococcus pyogenes* Cas9 (SpCas9) open reading frame (ORF) that was codon optimized for rice and cloned it into a binary vector for *Agrobacterium*-mediated transformation (Fig. 1C). In this construct, Cas9 was driven by the constitutive double 35S *Cauliflower mosaic virus* promoter (2×P35S). The OsU3 promoter was used to express the sgRNA. Cas9 and sgRNA expression cassettes were cloned into independent binary vectors and transformed sequentially into rice calli (see the Materials and Methods).

### Detection of on-target mutation in Cas9, sgRNA-expressing calli

**Fig. 2:**
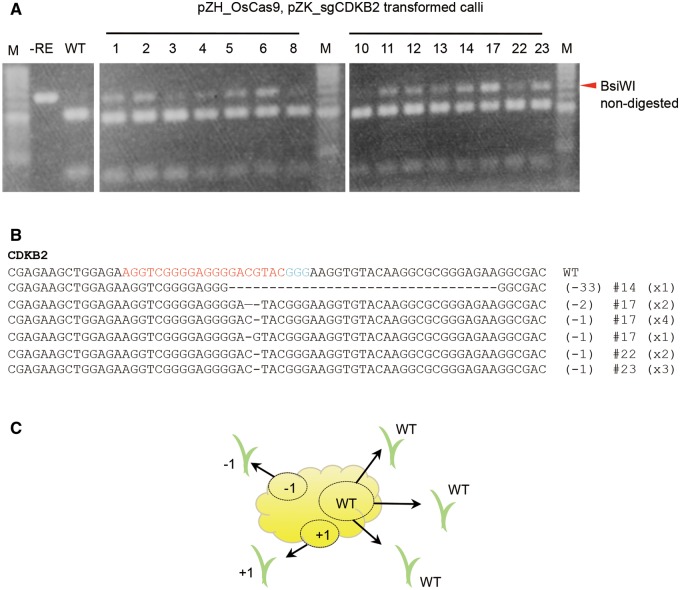
Detection of mutations in the *CDKB2* gene in Cas9-, sgRNA-expressing calli. (A) CAPS analysis of the *CDKB2* locus. DNA extracted from independent pZK_sgCDKB2-transformed calli was subjected to PCR and subsequent *Bsi*WI restriction enzyme digestion. M, marker; –RE, PCR product without restriction enzyme reaction; WT, *Bsi*WI-digested PCR product of wild-type rice DNA. (B) Representative sequences of mutant alleles identified from Cas9-, sgRNA-expressing calli. The wild-type sequence is shown at the top with the PAM sequence highlighted in cyan and the target sequence in red. Dashes indicate deleted bases. The net change in length is noted to the right of each sequence (+, insertion; – deletion). The number of clones representing each mutant allele is shown in brackets. (C) Schematic representation of the chimeric state of multiply mutated and non-mutated cells in clonally propagated transgenic callus. The high proportion of mutated cells makes it easy to obtain mutated plants. Because mutations occur independently in single cells, various mutants can be obtained from a single Cas9, sgRNA transgenic line.

The expected cleavage site lies within the recognition sequence of the restriction enzyme *Bsi*WI (Fig. 1B), so that this restriction site will be disrupted if CRISPR/Cas9 has cleaved the target sequence successfully. Thus, cleaved amplified polymorphic sequences (CAPS) were used to detect mutations in the target region. To select mutated callus from clonally propagated transgenic calli, we conducted CAPS analysis using DNA extracted from independent transgenic calli 1 month after sgRNA transformation. PCR products of 650 bp in length without mutations in the *Bsi*WI recognition site should be cleaved into 163 and 487 bp fragments by digestion with *Bsi*WI. CAPS analysis revealed that mutations were induced in almost all transgenic lines (Fig. 2A). Next, DNA sequencing analysis was conducted on cloned PCR products from four different lines (#14, #17, #22 and #23) to calculate mutation frequency accurately. We found that 4% (1/24), 29% (7/24), 8% (2/24) and 25% (6/24) of cloned PCR products contained mutations in *CDKB2* in lines #14, #17, #22 and #23, respectively. Details of detected mutations are shown in Fig. 2B. A small deletion at the site of cleavage was the mutation found most often, but large deletions >30 bp were also detected. Because non-mutated and various mutated PCR products were detected from the same clonally propagated transgenic callus (#17), each Cas9/sgRNA-expressed callus seems to be a chimera of mutated and non-mutated cells (Fig. 2C).

### Obtaining regenerated rice plants with on-target mutations in the *CDKB2* gene

**Fig. 3:**
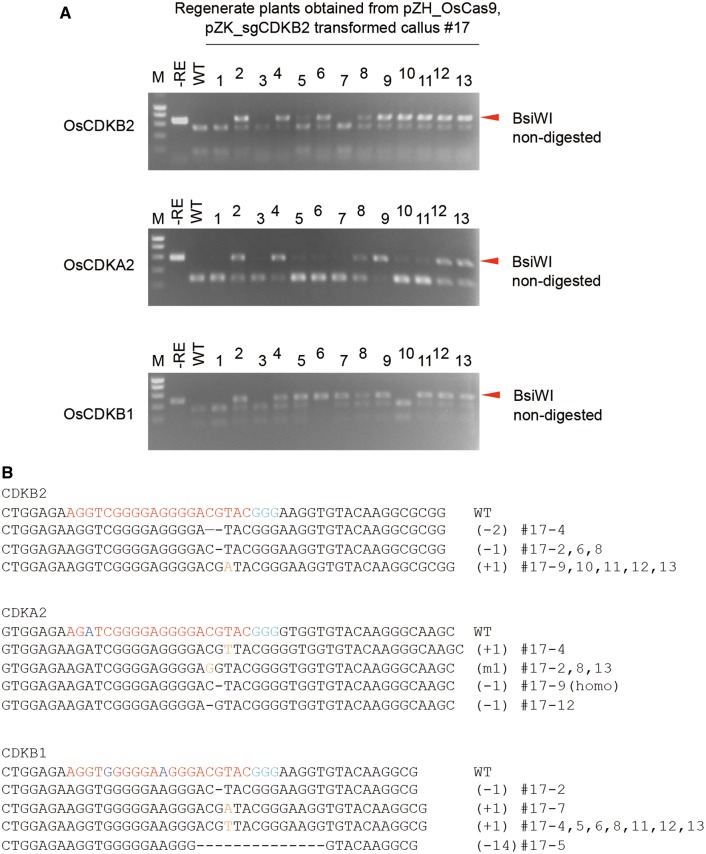
On- and off-target mutation in regenerated plants. (A) CAPS analysis of the *CDKB2*, *CDKA2* and *CDKB1* loci. DNA extracted from independent regenerated plants obtained from pZH_OsCas9, pZK_sgCDKB2-transformed callus #17 was subjected to PCR and subsequent *Bsi*WI restriction enzyme digestion. (B) Representative sequences of mutant alleles identified from regenerated plants. Mismatched bases to the target sequence are highlighted in blue in the wild-type sequence. Substituted and inserted bases are highlighted in orange.

The proportion of mutated cells in clonally propagated transgenic calli affects the ease of acquisition of mutated plants upon regeneration (Fig. 2C). Thus, we transferred highly mutated transgenic cell lines (calli) identified by CAPS analysis (Fig. 2A) to regeneration medium. In the case of #17, 10 out of 13 regenerated plants had a mutation at the target sequence in the *CDKB2* gene (Fig. 3A). When PCR products of mutated plants were sequenced, three types of mutation were detected (Fig. 3B). Since only a single mutation pattern was detected in DNA extracted from a mixture of several tillers of independent regenerated plants, regenerated plants seemed to originate from a single or a few cells, and the chimeric mixture of non-mutated and various kinds of mutated cells in a single callus might be resolved during the process of regeneration.

### Simultaneous mutations in *CDKA2* and *CDKB1* in regenerated plants

Because the 20 bp target sequence in pZK_sgCDKB2 has homology to *CDKA1*, *CDKA2* and *CDKB1* (Fig. 1B) to different degrees, off-target mutations in these genes in regenerated plants of #17 were analyzed by CAPS analysis using *Bsi*WI digestion. This analysis revealed that a large number of regenerated plants containing on-target mutations in *CDKB2* also had mutations in *CDKA2* and *CDKB1* genes (Fig. 3A). On the other hand, no mutation was detected in *CDKA1*, which possessed three mismatches at 6, 14 and 17 bp 5′ upstream of the PAM sequence (data not shown) in all plants analyzed. Combinations of mutated genes in regenerated plants are shown in Table 1. To investigate these mutations in more detail, we sequenced the off-target site in each regenerated plant. Sequencing analysis revealed that variations of on- and off-target mutation differed among the regenerated plants even if the combination of mutated genes was the same (Fig. 3B). Details of on- and off-target mutations in regenerated plants obtained from other transgenic lines are shown in Table 2 and Supplementary Fig. S3. In 29 regenerated plants, single mutants of *CDKB2*, *CDKA2* and *CDKB1*, double mutants of *CDKB2* and *CDKA2*, *CDKB2* and *CDKB1*, and triple mutants of these three genes existed. The ratios of plants with mutated *CDKB2*, *CDKA2* and *CDKB1* genes were 20/29, 18/29 and 16/29, respectively. Because no plant showed more than two variations of mutated sequences at each gene, novel on- and off-target mutations were not induced after regeneration in our study.

**Table 1:** Mutations in *CDKA* and *CDKB* sequences in regenerated plants obtained from pZH_OsCas9, pZK_sgCDKB2-transformed callus #17

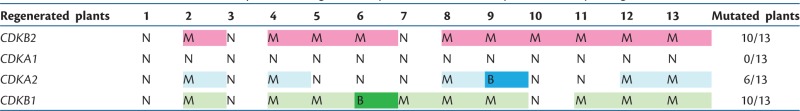

N, no mutation; M, monoallelic mutation; B, biallelic mutation.

**Table 2:** Mutations in *CDKA* and *CDKB* genes in regenerated plants obtained from pZH_OsCas9, pZK_sgCDKB2-transformed calli #5, 11, 12 and 13

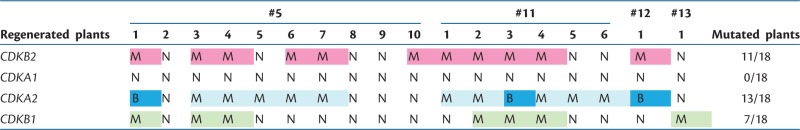

N, no mutation; M, monoallelic mutation; B, biallelic mutation.

On the other hand, off-target sites can be predicted computationally. According to the free web tool CRISPR-P (Lei et al. 2014; http://cbi.hzau.edu.cn/crispr/), *CDKA2* and *CDKB1* are the two most likely potential off-target candidates and *CDKA1* is the 10th most likely potential candidate. We also analyzed the existence of third, fifth and ninth off-target candidates in regenerated plants by CAPS analysis, but no regenerated plants containing mutations in these off-target candidate loci were detected (Supplementary Fig. S4).

## Discussion

As previous studies have reported, off-target mutations were detected in rice plants obtained from transgenic calli of Cas9 and sgRNA expression vectors. An off-target mutation in *CDKA2*, which has a homologous sequence with just a 1 bp mismatch 17 bp 5′ upstream of the PAM sequence, was detected frequently. This result is not surprising because a 1 bp mismatch from the out-of-seed sequence seems to be acceptable (Supplementary Fig. S5; Fu et al. 2013, Hsu et al. 2013, Pattanayak et al. 2013). In addition, although not occurring in *CDKA2*, mutations in *CDKB1* were still detected frequently. Furthermore, biallelic mutations were detected in the off-target genes *CDKA2* and *CDKB1*, but not in the on-target gene, *CDKB2*. In a previous study, we revealed that knockdown of *CDKB2* in rice cells induces endomitosis (Endo *et al.*, 2012). Thus, we expect that *CDKB2* is essential for the progression of mitosis and that this may be the reason why no biallelic mutant of the *CDKB2* gene was obtained in this study. Because *CDKB2*, *CDKA2* and *CDKB1* are located on chromosomes 8, 2 and 1, respectively, and almost all mutations detected in regenerated plants were monoallelic, single, double and triple mutants of these three genes with different mutations might be obtained in the progeny of regenerated plants. Because no multigene knockout of these fundamental cell cycle regulators has been created before, these mutated rice plants are attractive materials with which to identify the function of each gene and to study the redundancy of CDK genes.

However, off-target mutation is sometimes considered troublesome. If the genome sequence of the subject is available, selecting a target sequence with high specificity is important for avoiding off-target mutations. In addition, we have found that prolonged culture of Cas9- and sgRNA-expressing calli accumulates on-target mutations (M. Mikami et al. unpublished results). Because on-target mutations may precede off-target mutations, and novel mutations were not induced after regeneration, a short selection period for calli at the stage of redifferentiation may be effective in preventing off-target mutations. To reduce the risk of off-target mutation more actively, utilization of paired Cas9 nickase, composed of D10A Cas9 and two sgRNAs, which generate two single-strand breaks or nicks on different DNA strands, seems to be effective (Ran et al. 2013). In plants, Fauser et al. (2014) reported enhanced intrahomologous recombination following application of paired Cas9 nickase to Arabidopsis, which suggests the induction of DNA breaks at the target site.

Previously, RNA interference (RNAi)-mediated knockdown was the strategy most commonly used for depleting cells of a gene product of interest. However, RNAi usually does not shut off a gene completely. In contrast, artificial nuclease-mediated gene modification changes the genetic code, typically causing a ‘knockout’, or complete elimination, of gene function. The CRISPR/Cas9 system is a very convenient artificial nuclease because just 20 bp in the sgRNA can specify the cleavage points. Simultaneous expression of multiple sgRNAs is obviously an effective measure for creating multigene knockout plants. However, knockout of multigenes by a single sgRNA is also useful because the entire genome information is not always available; many crop species are relatively recent allopolyploids, resulting from interspecific hybridization, and the genome-wide analysis of polyploidy crops has lagged behind that of diploid crops and other model organisms. Off-target mutation of CRISPR/Cas9 due to mismatch admissibility may be useful for disruption of duplicated genes even if only poor or partial genomic information is available.

## Materials and Methods

### Vector construction

The Cas9 expression vector, pZH_OsCas9, was constructed as follows: (i) the Cas9 ORF was codon optimized for rice by Fasmac (Kanagawa). (ii) Connected sequences of the rice alcohol dehydrogenase (ADH) 5′-untranslated region (UTR), codon-optimized Cas9 and pea rbcS3A (pea3A) terminator sequence were synthesized flanked by *Xba*I and *Pac*I sites. (iii) This synthesized fragment (OsADH5′UTR::OsCas9::Tpea3A) was cloned downstream of the double *Cauliflower mosaic virus* 35S promoter (2×P35S) in pE(L3-L2) using *Xba*I and *Pac*I sites. (iv) The 2×P35S::OsADH5′UTR::OsCas9::TpeasA fragment was digested with *Asc*I and *Pac*I, and cloned into pZH with a hygromycin resistance cassette (2×P35S ::HPT::T35STnos) (Kuroda *et al.*, 2010).

The guide RNA expression vector, pZK_sgCDKB2, was constructed as follows. (i) Connected sequence of the OsU3 promoter 20 bp target sequence, sgRNA scaffold and poly(T) was synthesized by Fasmac flanked by *Asc*I and *Pac*I sites. (ii) This synthesized fragment was cloned into pZK with a kanamycin resistance cassette [2xP35S::NPTII::T35S] using *Asc*I and *Pac*I sites (Kuroda et al. 2010).

### Transformation of Cas9, sgRNA expression vectors

*Agrobacterium*-mediated transformation of rice (*O. sativa* L. cv. Nipponbare) was performed as described (Toki 1997, Toki et al. 2006). After co-cultivation of *Agrobacterium* carrying the Cas9 expression vector, pZH_OsCas9, with rice scutellum-derived calli (pre-cultured for 3 weeks) for 3 d, infected calli were transferred to fresh callus induction medium (Toki 1997) containing 50 mg l^–1^ hygromycin B (Wako Pure Chemicals), and 25 mg l^–1^ meropenem (Wako Pure Chemicals) to remove *Agrobacterium*. One month after hygromycin selection, proliferated calli were transferred to callus induction medium without meropenem and cultured for 1 week before transformation of the sgRNA expression vector, pZK_sgCDKB2. pZH_OsCas9-transformed calli were gathered and subjected to a second transformation using pZK_sgCDKB2. After 3 d of co-cultivation, pZK_sgCDKB2-transformed calli were transformed to fresh callus induction medium containing 35 mg l^–1^ G418 (Wako Pure Chemicals) and 25 mg l^–1^ meropenem (Wako Pure Chemicals).

### CAPS analysis

DNA was extracted from calli or regenerated plants. *CDKB2*, *CDKA2* and *CDKB1* loci were PCR amplified using the following primers: CDKB2-F, 5′-AAACCCTAAATCCACGCGCATTCCACACCA-3′; CDKB2-R, 5′-TGGCAGAAAGCAACGCCCTTGCAGAGCTGGT-3′; CDKA2-F, 5′-ATGCCACAAGCCCAACCCAATTCATCCCCA-3′; CDKA2-R 5′-TGCAGCCTGCACGGCACAATCCAAATTCCCA-3′; CDKB1-F, 5′-ACGCTCCTCCCCCATTTCAAATC-3′; and CDKB1-R, 5′-AGAAGCGGAGAAGACACGGGATAATCAGGCA-3′.

PCR products were subjected to a *Bsi*WI restriction enzyme reaction and analyzed by agarose gel electrophoresis.

### Sequencing analysis

PCR products used for restriction fragment length polymorphism analysis were cloned into pCR-BluntII-TOPO (Invitrogen) and subjected to sequencing analysis using an ABI3130 sequencer (Applied Biosystems).

## Supplementary data

Supplementary data are available at PCP online.

## Funding

This work was supported by the National Institute of Agrobiological Sciences Strategic Research Fund.

## Supplementary Material

Supplementary Data

## Abbreviations

CAPS: cleaved amplified polymorphic sequence
Cas9: CRISPR-associated endonuclease 9
CDK: cyclin-dependent kinase
CRISPR: clustered regularly interspaced short palindromic repeat
ORF: open reading frame
PAM: protospacer-adjacent motif
sgRNA: single-guide RNA
