# Supplementary material for: Multigene Knockout Utilizing Off-Target Mutations of the CRISPR/Cas9 System in Rice
Source: Plant Cell Physiol. 2014 Nov 11;56(1):41–7. doi: 10.1093/pcp/pcu154 (PMC4301742; doi:10.1093/pcp/pcu154)
Supplement: Supplementary Data [file supp_56_1_41__index.html]

Multi-gene knockout utilizing off-target mutations of the CRISPR/Cas9 system in rice — Multigene Knockout Utilizing Off-Target Mutations of the CRISPR/Cas9 System in Rice — Multigene Knockout Utilizing Off-Target Mutations of the CRISPR/Cas9 System in Rice — Supplementary Data 

# Multigene Knockout Utilizing Off-Target Mutations of the CRISPR/Cas9 System in Rice

## Supplementary Data

files

**Files in this Data Supplement:**

- Supplementary Data - pdf file
