# Supplementary material for: Multigene Knockout Utilizing Off-Target Mutations of the CRISPR/Cas9 System in Rice
Source: Plant Cell Physiol. 2014 Nov 11;56(1):41–7. doi: 10.1093/pcp/pcu154 (PMC4301742; doi:10.1093/pcp/pcu154)
Supplement: Supplementary Data [file supp_pcu154_pcp-2014-e-00488-File006.pdf]

Supplementary Figure S1. Alignment of amino acid sequences of OsCDKAs and OsCDKB2.

```
OsCDKA1   MEQYEKEEKIGEGTYGVVYRARDKVTNETIALKKIRLEQEDEGVPSTAIREISLLKEMHH  60
OsCDKA2   MEQYEKVEKIGEGTYGVVYKGKHRHTNETIALKKIRLEQEDEGVPSTAIREISLLKEMQH  60
OsCDKB1   MEKYEKLEKVGEGTYGKVYKAQDRATGQLVALKKTRLEMDEEGIPPTALREISILRLLSQ  60
OsCDKB2   MDLYEKLEKVGEGTYGKVYKAREKATGRIVALKKTRLPEDDEGVPPTALREVSLLRMLSQ  60

OsCDKA1   G-NIVRLHDVIHSE-----KRIYLVFEYLDLDLKKFMDSCPEFA----KNPTLIKSYLYQ 110
OsCDKA2   R-NIVRLQDVVHKE-----KCIYLVFEYLDLDLKKHMDSSPDF-----KNHRIVKSFLYQ 109
OsCDKB1   SLYVVRLLSVEQAT-KNGKPVLYLVFEFLDTDLKKFVDAYRKGPNPRPLPTNVIKSFLYQ 119
OsCDKB2   DSHVVRLLDLKQGQNKEGQTILYLVFEYMDTDLKKFIRAHRQNLQKIPVPT--VKILMYQ 118

OsCDKA1   ILRGVAYCHSHRVLHRDLKPQNLLIDRRTNALKLADFGLARAFGIPVRTFTHEVVTLWYR 170
OsCDKA2   ILRGIAYCHSHRVLHRDLKPQNLLIDRRTNSLKLADFGLARAFGIPVRTFTHEVVTLWYR 169
OsCDKB1   LCKGVAHCHGHGVLHRDLKPQNLLVDKEKGILKIADLGLGRAFTVPMKSYTHEIVTLWYR 179
OsCDKB2   LCKGVAFCHGRGVLHRDLKPHNLLMDRKTMALKIADLGLSRSFTVPLKKYTHEILTLWYR 178

OsCDKA1   APEILLGSRQYSTPVDMWSVGCIFAEMVNQKPLFPGDSEIDELFKIFRVLGTPNEQSWPG 230
OsCDKA2   APEILLGARHYSTPVDMWSVGCIFAEMVNQKPLFPGDSEIDELFKIFSIMGTPNEETWPG 229
OsCDKB1   APEVLLGSTHYSTGVDIWSVGCIFAEMVRRQALFPGDSELQQLLHIFRLLGTPTEEQWPG 239
OsCDKB2   APEVLLGAAHYSTPVDIWSVGCIFAELATNQPLFAGDSEVQQLLHIFKLLGTPNEQVWPG 238

OsCDKA1   VSSLPDYKSAFPKWQAQDLATIVPTLDPAGLDLLSKMLRYEPNKRITARQALEHEYFKDL 290
OsCDKA2   VASLPDYISTFPKWPSVDLATVVPTLDSSGLDLLSKMLRLDPSKRINARAALEHEYFKDL 289
OsCDKB1   VTDLRDWH-EFPQWKPQILERQVPSLEPEGVDLLSKMLQYNPANRISAKAAMEHPYFDSL 298
OsCDKB2   VSKLPNWH-EYPQWNPSKVSDLVHGLDADALDLLEKMLQYEPSKRISAKKAMEHPYFNDV 297

OsCDKA1   EMVQ- 294
OsCDKA2   EVA-- 292
OsCDKB1   DKSQF 303
OsCDKB2   NKELY 302
```

Amino acids sequence coded by 20 bp target sequence in sgRNA is underlined.

Supplementary Figure S2   Alignment of cDNAs of OsCDKAs and OsCDKB2.

```
OsCDKA1         ATGGAGCAGTACGAGAAGGAGGAGAAGATTGGGGAGGGCACGTACGGGGTGGTGTACAGG 60
OsCDKA2         ATGGAGCAGTACGAGAAGGTGGAGAAGATCGGGGAGGGGACGTACGGGGTGGTGTACAAG 60
OsCDKB1         ATGGAGAAGTACGAGAAGCTGGAGAAGGTGGGGGAAGGGACGTACGGGAAGGTGTACAAG 60
OsCDKB2         ATGGACCTGTACGAGAAGCTGGAGAAGGTCGGGGAGGGGACGTACGGGAAGGTGTACAAG 60
                *****   **********  ******* * ***** ** *********  ******** *

OsCDKA1         GCGCGGG-ACAAGGTCACCAACGAGACGATCGCGCTCAAGAAGATCCGGCTTGAGCAGGA 119
OsCDKA2         G-GCAAGCACCGGCATACCAACGAGACGATCGCGCTCAAGAAGATCCGCCTGGAGCAGGA 119
OsCDKB1         GCGCAGG-ACAGGGCGACGGGGCAGCTGGTGGCGCTGAAGAAGACGAGGCTGGAGATGGA 119
OsCDKB2         GCGCGGG-AGAAGGCGACGGGGCGGATCGTGGCGCTGAAGAAGACGCGGCTGCCGGAGGA 119
                * **  * *   *   **      *    *  ***** *******   * **   *  ***

OsCDKA1         GGATGAGGGCGTCCCCTCCACCGCAATCCGCGAGATCTCGCTCCTCAAGGAGATGCATCA 179
OsCDKA2         GGACGAGGGCGTCCCCTCCACCGCCATCCGCGAGATCTCGCTGCTCAAGGAGATGCAGCA 179
OsCDKB1         CGAGGAGGGGATCCCGCCCACCGCGCTGCGCGAGATCTCCATCCTCAGGCTGCTCTCCCA 179
OsCDKB2         CGACGAGGGCGTGCCGCCGACGGCGCTCAGGGAGGTGTCGCTGCTGCGGATGCTGTCGCA 179
                 ** *****  * **  * ** **  *  * *** * **  * **   *  * *    **

OsCDKA1         CG-GC---AACATCGTCAGGTTACACGATGTTATCCAC------AGTGAGAAGCGCA--- 226
OsCDKA2         TC-GC---AACATCGTCAGGCTGCAGGACGTCGTGCAC------AAGGAGAAATGCA--- 226
OsCDKB1         GTCGCTC-TACGTCGTCCGCCTCCTCTCCGTCGAGCAGGCC---ACCAAGAACGGCAAGC 235
OsCDKB2         GG-ACTCGCACGTGGTGCGCCTGCTGGACCTCAAGCAGGGGCAGAACAAGGAGGGCCAGA 238
                    *    ** * **  *  * *      *    **       *   ** *  **

OsCDKA1         ------TATATCTTGTCTTTGAGTATCTGGATCTGGACCTAAAGAAGTTCATGGACTCTT 280
OsCDKA2         ------TATACCTCGTCTTCGAGTACCTCGACCTTGACCTCAAGAAGCACATGGACTCAT 280
OsCDKB1         CCGTCCTCTACCTCGTCTTCGAGTTCCTCGACACCGACCTCAAGAAGTTCGTCGACGCCT 295
OsCDKB2         CCATCCTCTACCTCGTCTTCGAGTACATGGACACCGACCTCAAGAAGTTCATCCGCGCCC 298
                      * ** ** ***** ****   * **    ***** ******  * *   * *

OsCDKA1         GTC--CAGAGT-TTGCGAAAAACCCCACTTTAA---------TTAAGTCATATCTCTATC 328
OsCDKA2         CCC--CGGATT-TCAAGAACCACCGCA---TAG---------TCAAATCGTTCCTCTACC 325
OsCDKB1         ACCGCAAGGGCCCCAACCCTCGCCCCCTCCCCACCAACGTCATCAAGAGCTTCTTGTATC 355
OsCDKB2         ACCGCCAGAATCTCCAGAAGATCCCCGTGCCCACCG------TCAAGATCCTGATGTACC 352
                  *    *              ** *                * **        * ** *

OsCDKA1         AGATACTCCGCGGCGTTGCTTACTGTCATTCTCATAGAGTTCTTCATCGAGATTTGAAAC 388
OsCDKA2         AGATTCTCCGGGGCATTGCGTACTGCCACTCGCACCGTGTTCTCCACCGAGATTTGAAGC 385
OsCDKB1         AGTTATGCAAAGGAGTCGCACATTGCCATGGCCATGGTGTCCTTCACCGTGATTTAAAGC 415
OsCDKB2         AGCTCTGCAAGGGCGTTGCTTTCTGCCATGGCCGCGGTGTGCTGCACCGTGACCTCAAGC 412
                ** *   *   **  * **    ** **    *   * ** ** ** ** **  * ** *

OsCDKA1         CTCAGAATTTATTGATAGATCGGCGTACTAATGCACTGAAGCTTGCAGACTTTGGTTTAG 448
OsCDKA2         CCCAGAACCTGCTGATAGATCGGCGTACCAACTCATTGAAGCTCGCGGACTTTGGGTTGG 445
OsCDKB1         CACAAAACCTGTTGGTCGACAAGGAAAAGGGGATATTGAAAATTGCTGATCTTGGGCTAG 475
OsCDKB2         CGCATAACCTGCTCATGGACCGCAAGACCATGGCGCTCAAGATCGCCGACCTCGGGCTCA 472
                * ** **  *  *  * **       *         * **  * ** **  * **  *

OsCDKA1         CCAGGGCATTTGGAATTCCTGTCCGCACGTTTACTCACGAGGTTGTAACCTTGTGGTATA 508
OsCDKA2         CCAGGGCATTTGGCATTCCTGTCCGGACATTTACTCACGAGGTGGTGACATTGTGGTATA 505
OsCDKB1         GTAGGGCGTTCACTGTTCCTATGAAAAGCTACACACATGAGATTGTGACTCTTTGGTACA 535
OsCDKB2         GCCGCTCGTTCACTGTCCCTCTCAAGAAGTACACCCACGAGATCCTGACGCTGTGGTACA 532
                   *  * **     * *** *    *  *  ** ** *** *  * **  * ***** *

OsCDKA1         GAGCTCCAGAGATCCTTCTTGGATCAAGGCAGTATTCTACACCAGTTGATATGTGGTCAG 568
OsCDKA2         GAGCACCTGAAATTCTTCTTGGTGCAAGGCATTATTCCACCCCTGTTGACATGTGGTCAG 565
OsCDKB1         GAGCTCCTGAAGTTTTGCTTGGATCAACACATTACTCAACCGGTGTTGACATTTGGTT-- 593
OsCDKB2         GGGCTCCCGAGGTTCTTCTTGGCGCGGCACACTACTCCACTCCGGTTGACATCTGGTCTG 592
                * ** ** **  *  * *****  *    ** ** ** **    ***** ** ****
```

```
OsCDKA1         TTGGTTGTATCTTTGCAGAAATGGTGAACCAGAAACCACTGTTCCCTGGTGATTCTGAGA 628
OsCDKA2         TTGGTTGCATTTTTGCTGAAATGGTGAATCAGAAGCCACTATTTCCTGGAGATTCTGAGA 625
OsCDKB1         --------------------------------------------GTTGGGAACTCC---- 605
OsCDKB2         TGGGCTGCATTTTTGCTGAGTTGGCCACTAACCAACCTCTTTTCGCTGGAGATTCCGAGG 652
                                                              ***  * **

OsCDKA1         TTGATGAATTATTTAAGATATTCAGGGTACTAGGAACTCCAAATGAACAAAGTTGGCCAG 688
OsCDKA2         TTGATGAACTCTTTAAGATTTTCAGTATTATGGGCACTCCAAATGAAGAAACTTGGCCAG 685
OsCDKB1         ---------------------------TACTGAG----------GAGCAG---TGGCCTG 625
OsCDKB2         TTCAGCAGCTTCTGCACATTTTCAAGTTGCTGGGCACCCCAAATGAGCAAGTTTGGCCAG 712
                                           *  *  *          **  *    ***** *

OsCDKA1         GAGTTAGCTCATTACCTGACTACAAGTCTGCTTTCCCCAAGTGGCAAGCACAGGATCTTG 748
OsCDKA2         GTGTTGCTTCACTACCTGACTACATATCAACTTTCCCAAAGTGGCCATCTGTGGATCTTG 745
OsCDKB1         GAGTAACTGATTTGAGGGACTG---GCATGAGTTTCCACAGTGG-AAGCCACAGATTTTA 681
OsCDKB2         GAGTGAGCAAGCTACCTAACTG---GCACGAGTACCCCCAGTGG-AATCC-----CTCGA 763
                * **        *     ***           *  **  *****  * *

OsCDKA1         CAAC-TATTGTCCCTACTCTTG-----ACCCTGCTGGTTTGGACCTTCTCTCTAAAATGC 802
OsCDKA2         CAAC-CGTGGTCCCAACACTTG-----ATTCTTCAGGACTCGATCTTCTCTCTAAAATGC 799
OsCDKB1         GAACGTCAAGTCCCATCATTGG-----AGCCTGAAGGAGTTGACCTTTTATCGAAAATGC 736
OsCDKB2         AAGTGTCTGATCTTGTCCATGGTCTCGACGCTGATGCTCTTGATCTTCTTGAGAAAATGC 823
                 *        **    *  * *     *  **   *   * ** *** *    *******

OsCDKA1         TTCGGTACGAGCCAAACAAAAGGATCACAGCTAGACAGGCTCTTGAGCATGAATACTTCA 862
OsCDKA2         TCCGTTTAGATCCAAGCAAAAGAATCAATGCCCGTGCTGCCCTCGAGCACGAGTACTTCA 859
OsCDKB1         TCCAGTATAACCCAGCAAATCGGATCTCAGCAAAGGCTGCTATGGAACACCCCTACTTCG 796
OsCDKB2         TGCAGTACGAGCCGTCGAAGCGGATCTCTGCGAAGAAGGCCATGGAGCATCCCTACTTCA 883
                * *  *   * **    **  * ***   **       **  * ** **    ******

OsCDKA1          AGGACCTTGAGATGG------TACAATGA 885
OsCDKA2          AGGACCTGGAAGTGG------CGTAG--- 879
OsCDKB1          ACAGCCTCGACAAGTCCCAGTTCTAG--- 822
OsCDKB2          ACGACGTGAACAAGG---AGCTCTACTGA 909
                *  * *  *  *         *
```

PAM sequence is shown in cyan and 20bp target sequence is shown in red.

Supplementary Figure S3. Mutations in CDKAs and CDKBs in regenerated plants obtained from pZH_OsCas9, pZK_sgCDKB2 transformed calli #5,11,12,13.

```
CDKB2
CGAGAAGCTGGAGAAGGTCGGGGAGGGGACGTACGGGAAGGTGTACAAGGCGCGG   WT
CGAGAAGCTGGAGAAGGTCGGGGAGGGGAC-TACGGGAAGGTGTACAAGGCGCGG   (-1) #5-1,3,#11-2,3,4,#12-1
CGAGAAGCTGGAGAAGGTCGGGGAGGGGA--TACGGGAAGGTGTACAAGGCGCGG   (-2) #11-2
CGAGAAGCTGGAGAAGGTCGGGGAGGGGACGATACGGGAAGGTGTACAAGGCGCGG  (+1) #5-4,6,7,10,#11-1


CDKA2
CGAGAAGGTGGAGAAGATCGGGGAGGGGACGTACGGGGTGGTGTACAAGGGCAAG   WT
CGAGAAGGTGGAGAAGATCGGGGAGGGGACG----GGGTGGTGTACAAGGGCAAG   (-4) #11-3,4
CGAGAAGGTGGAGAAGATCGGGGAGGGGAC-TACGGGGTGGTGTACAAGGGCAAG   (-1) #5-1,3,4,#11-2,3,5,6,#12-1
CGAGAAGGTGGAGAAGATCGGGGAGGGGACG-ACGGGGTGGTGTACAAGGGCAAG   (-1) #5-5
CGAGAAGGTGGAGAAGATCGGGGAGGGGACGATACGGGGTGGTGTACAAGGGCAAG  (+1) #5-6,7,#11-1


CDKB1
CGAGAAGCTGGAGAAGGTGGGGGAAGGGACGTACGGGAAGGTGTACAAGGCG      WT
CGAGAAGCTGGAGAAGGTGGGGGAAGGGACGGTACGGGAAGGTGTACAAGGCG     (+1) #5-1
CGAGAAGCTGGAGAAGGTGGGGGAAGGGAC-TACGGGAAGGTGTACAAGGCG      (-1) #11-2,3,4
CGAGAAGCTGGAGAAGGTGGGGGAAGGGACGTTACGGGAAGGTGTACAAGGCG     (+1) #13-1
CGAGAAGCTGGAGAAGGTGGGGGAAGGG-------------TGTACAAGGCG      (-13) #5-3
CGAGAAGCTGGAGAAGGTGGGGGAAGGGACGTACGGGAAGGGGTACAAGGGG       (m2) #5-4
```

Representative sequences of mutant alleles identified from Cas9, sgRNA expressed calli. The wild type sequence is shown at the top with PAM sequence highlighted in cyan and the target sequence in red. Dash, deleted bases. The net change in length is to the right of each sequence (+, insertion; - deletion).

Supplementary Figure S4. Potential off target sites predicted by CRISPR-P.

	AGGTCGGGGAGGGGACGTACGGG	On-target	(Chr.8 CDKB2)
1	AGATCGGGGAGGGGACGTACGGG	Detected	(Chr.2 CDKA2)
2	AGGTGGGGGAAGGGACGTACGGG	Detected	(Chr.1 CDKB1)
3	CGGGCGGCGTGGGGACGTACGAG	No-detected	(Chr.2)
4	GGGTCGGGGCGGGGACGTTCGGG		(Chr.1)
5	GGGCAGGGCAGGGGACGTACGGG	No-detected	(Chr.2)
6	GGGGGGAGGAGGGGACGTACAGG		(Chr.3)
7	AGGCGGTGAAGGGGACGTACCGG		(CHr.7)
8	AGGGAGGAGAGGAGACGTACAGG		(Chr.3)
9	AGGACGAGGAGGGGGCGTACGGG	No-detected	(Chr.3)
10	AGATTGGGGAGGGCACGTACGGG	No-detected	(Chr.3 CDKA1)
	*Bsi*WI		

The top 10 off-target sites predicted by CRISPR-P are listed. CDKA2, CDKB1 and CDKA1 are ranked as 1st, 2nd and 10th. Among 6 analyzed off-target candidates, mutations were detected in only top two candidates, CDKA2 and CDKB1.

Supplementary Figure S5. Acceptable mismatch in CRISPR/Cas9 system.

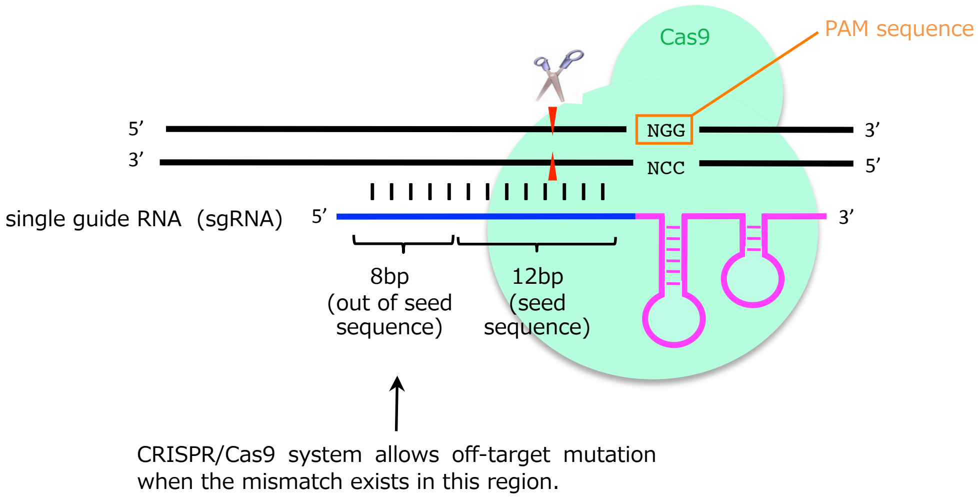

CRISPR/Cas9 system allows off-target mutation when mismatches exist in out of seed sequence.
